# Vestibular approach for thyroid surgery: a comprehensive review

**DOI:** 10.3389/fsurg.2024.1423222

**Published:** 2024-06-14

**Authors:** Luigi La Via, Antonio Zanghì, Andrea Cavallaro, Maria Di Vita, Antonino Maniaci, Salvatore Cocuzza, Alessandro Cappellani, Simone Di Majo

**Affiliations:** ^1^ Department of Anesthesia and Intensive Care, University Hospital Policlinico “G.Rodolico-San Marco”, Catania, Italy; ^2^ Department of Medical and Surgical Sciences and Advanced Technologies “GF Ingrassia”, University of Catania, Catania, Italy; ^3^ Centro di Ricerca in Chirurgia Delle Sindromi Malformative Complesse Della Transizione e dell’Età Adulta, University of Catania, Catania, Italy; ^4^ Department of General Surgery and Medical-Surgical Specialties, University of Catania, Catania, Italy; ^5^ Faculty of Medicine and Surgery, University of Enna “Kore”, Enna, Italy; ^6^ Residency Program in General Surgery, University of Catania, Catania, Italy

**Keywords:** transoral, endoscopic, thyroidectomy, neck, minimally invasive

## Abstract

The transoral endoscopic thyroidectomy vestibular approach (TOETVA) is an innovative technique in thyroid surgery. This review compiles current research on TOETVA, covering its development, anatomical challenges, techniques, selection of suitable patients, results, complications, and future advancements. We performed a comprehensive literature review on PubMed, EMBASE, and Cochrane databases for articles published up to 15th March 2024. The search strategy included a combination of terms focused on “vestibular approach” and “thyroidectomy”. The review underscores the necessity for preoperative planning and careful patient selection to reduce risks and enhance outcomes. It discusses the unique anatomical challenges of TOETVA, such as avoiding mental nerve damage and the complexities involved in creating a subplatysmal space. Outcomes of TOETVA, including surgical duration, complication rates, and recovery times, are compared favorably to traditional methods. The approach is particularly noted for high patient satisfaction and superior cosmetic results. Complications specific to TOETVA, like infection, bleeding, and potential harm to the recurrent laryngeal nerve, are recognized. Future research directions are discussed as well. In summary, TOETVA is a promising alternative for thyroidectomy with excellent cosmetic outcomes and patient satisfaction. Success relies on selective patient criteria, surgical expertise, and continuous research to refine the approach.

## 1 Introduction

In order to treat thyroid diseases, such as benign nodules, multinodular goiter, and cancers, thyroid surgery is a frequently performed treatment (1). Historically, a thyroidectomy necessitates a cervical incision, which can still be evident even after it is reasonably small and tucked into a skin fold to reduce visibility. This scar may cause psychological distress and unhappiness with the surgical result for certain patients, especially those who have a high concern for esthetic outcomes (2). There is a growing need for scarless surgical treatments, hence many systems for remote access have been developed. These include the retroauricular, transaxillary, and breast approaches, which, although minimizing scarring in the neck, may leave additional scars and can necessitate more extensive dissection (3). For thyroid surgery, the transoral vestibular approach (TOETVA) has emerged as a novel technique that offers a truly scar-free outcome by accessing the thyroid gland through natural orifices, specifically the oral cavity (4). This minimally invasive technique aligns with the principles of natural orifice transluminal endoscopic surgery (NOTES), aiming to reduce postoperative pain, improve cosmetic results, and potentially shorten recovery time (5). Initially introduced in Asia and subsequently adopted in various other regions, TOETVA has garnered attention for its innovative use of the oral vestibule as an entry point for endoscopic or robotic thyroid surgery (6). By avoiding external incisions, the technique provides an excellent cosmetic result, which is particularly appealing in cultures with a strong emphasis on aesthetic outcomes and in patients with a personal preference for avoiding neck scars (7). The transoral vestibular approach is not without challenges, however. It requires careful patient selection, meticulous surgical technique, and a comprehensive understanding of cervical anatomy from an unconventional perspective (8). Additionally, the approach has a distinct set of potential complications that surgeons must be adept at managing (9). This review will provide an in-depth analysis of the vestibular approach for thyroid surgery. It will explore the historical evolution of the technique, anatomical and technical considerations, patient selection criteria, clinical outcomes, complications, and the future direction of this surgical method. By synthesizing the current body of literature, this review aims to offer a valuable resource for surgeons considering the incorporation of TOETVA into their surgical practice and for patients seeking information on scarless thyroidectomy options.

## 2 Methods

We conducted a comprehensive literature search on PubMed, EMBASE, and Cochrane databases for articles published up to 15th March 2024. The search strategy included a combination of Medical Subject Headings (MeSH) and free-text terms to capture the concepts of “vestibular approach” and “thyroidectomy”. Search terms included “thyroid”, “surgery” “vestibular”, “thyroidectomy”, “minimally invasive”, “surgical”, “TOETVA”. Filters were applied to include articles in English and studies involving human subjects. Studies were included if they reported on the surgical procedure, patient selection, patient outcomes, during and after TOETVA. We considered observational studies, randomized controlled trials (RCTs), case-control studies, cohort studies, and cross-sectional studies. Reviews, meta-analyses, and clinical guidelines were also included to provide context and discuss current recommendations. Case reports, editorials, commentaries, and studies not specifically addressing TOETVA were excluded from the review. Two reviewers independently screened titles and abstracts for relevance, followed by a full-text review to determine eligibility. Discrepancies were resolved through discussion or consultation with a third reviewer.

## 3 Evolution of TOETVA

The development of the vestibular approach for thyroidectomy is a testament to the surgical community's ongoing effort to reduce the morbidity and improve the aesthetic outcomes associated with traditional thyroid surgery (10). The timeline of this evolution reflects advancements in surgical techniques, patient-centered care, and technological progress in medical instruments. The concept of performing thyroid surgery without a cervical scar was initially met with skepticism, given the complex anatomy and vital structures of the neck. However, the successful application of minimally invasive techniques in other surgical fields, particularly the NOTES procedures, inspired endocrine surgeons to explore similar approaches for thyroid surgery (11). The first documented TOETVA procedures originated in Asia, where there is a significant cultural emphasis on cosmetic outcomes and a preference for avoiding visible scars (12). Surgeons in South Korea and Thailand, led by Dr. W.Y. Chung and Dr. Angkoon Anuwong, respectively, were pioneers in developing and refining this technique (13). In 2016, Dr. Anuwong published the first series of TOETVA procedures, demonstrating the feasibility and safety of this approach (4). Early adopters of the vestibular approach reported on various aspects, including reduced postoperative pain, absence of visible scarring, and high levels of patient satisfaction with the cosmetic results (14). Following these initial successes, there was a concerted effort to standardize the technique, ensuring consistency and reproducibility of results (15). Key steps, such as incision placement in the oral vestibule, creation of the working space, and endoscopic dissection techniques, were meticulously defined (16). The use of specialized retractors and the adaptation of existing endoscopic equipment further refined the TOETVA procedure (17). The introduction of robotic surgery to TOETVA represented a significant milestone, allowing for enhanced dexterity, three-dimensional visualization, and improved ergonomics (18). Although robotic TOETVA is not as widespread due to cost and availability, it has demonstrated benefits in select cases, particularly for surgeons early in their learning curve (19). As the technique gained recognition, surgeons around the world began to adopt and adapt the TOETVA approach to their clinical practices (20). Variations emerged to accommodate different patient anatomies, surgical preferences, and healthcare settings (21). With the increasing number of TOETVA procedures being performed globally, a substantial body of evidence has accumulated, including comparative studies and meta-analyses (22). These studies have provided a more robust understanding of the indications, outcomes, and potential complications associated with the vestibular approach, contributing to its broader acceptance (23). The dissemination of the TOETVA technique has been facilitated by advancements in surgical training and simulation (24). Workshops, cadaveric courses, and virtual reality simulations have become valuable tools for training surgeons in the intricacies of the vestibular approach without compromising patient safety (25). Today, TOETVA is recognized as a viable and effective approach for select patients requiring thyroid surgery (26). Ongoing research focuses on extending the indications, refining patient selection criteria, and enhancing the safety profile of the procedure (15). Future directions also include the development of new instruments and technologies tailored to the unique requirements of transoral thyroid surgery (6). The benefit of transoral endoscopic thyroidectomy is the potential for scar-free surgery (27).

## 4 Surgical procedure

**Figure 1:**
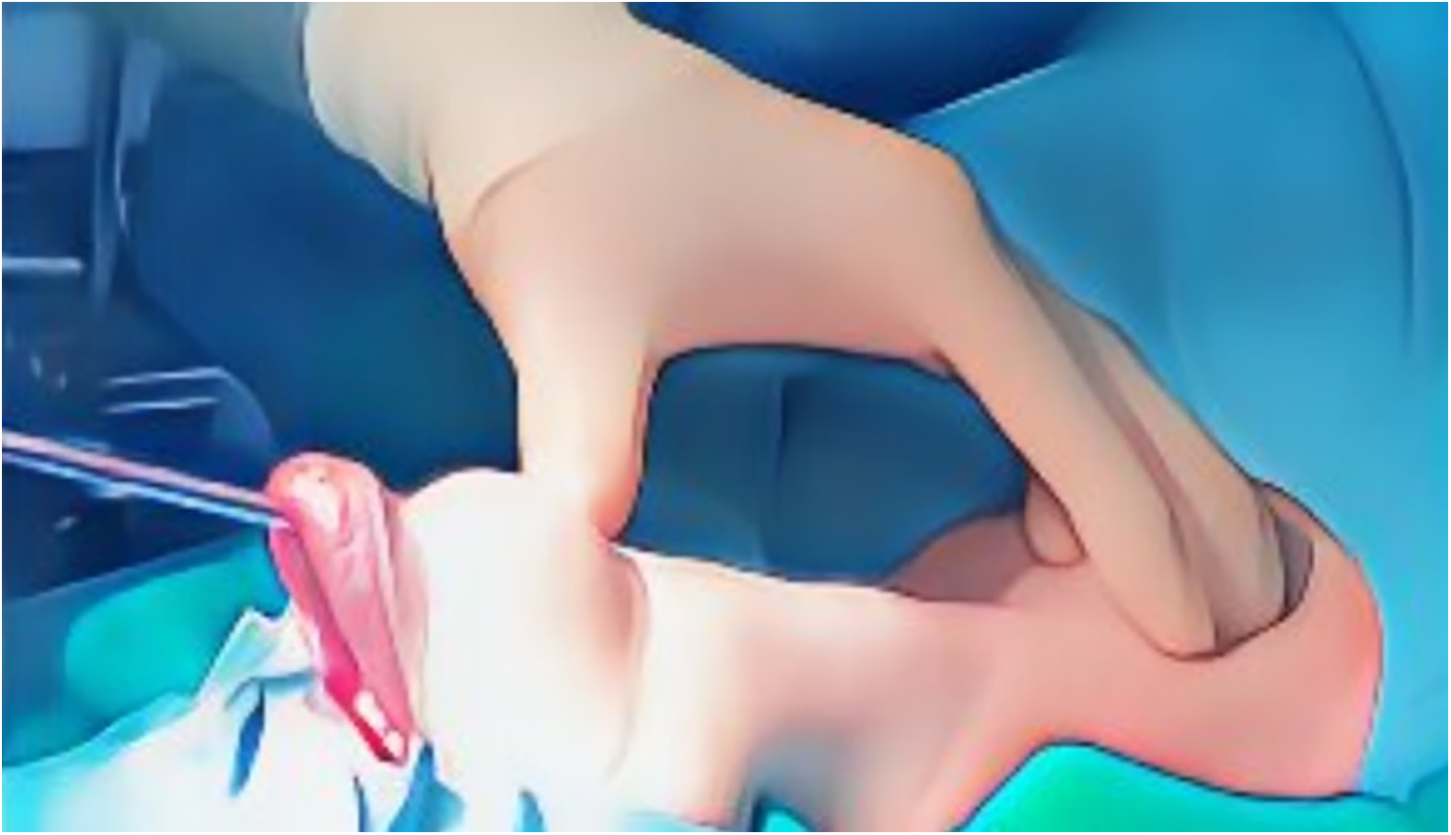
Surgical incision for TOETVA.

**Figure 2:**
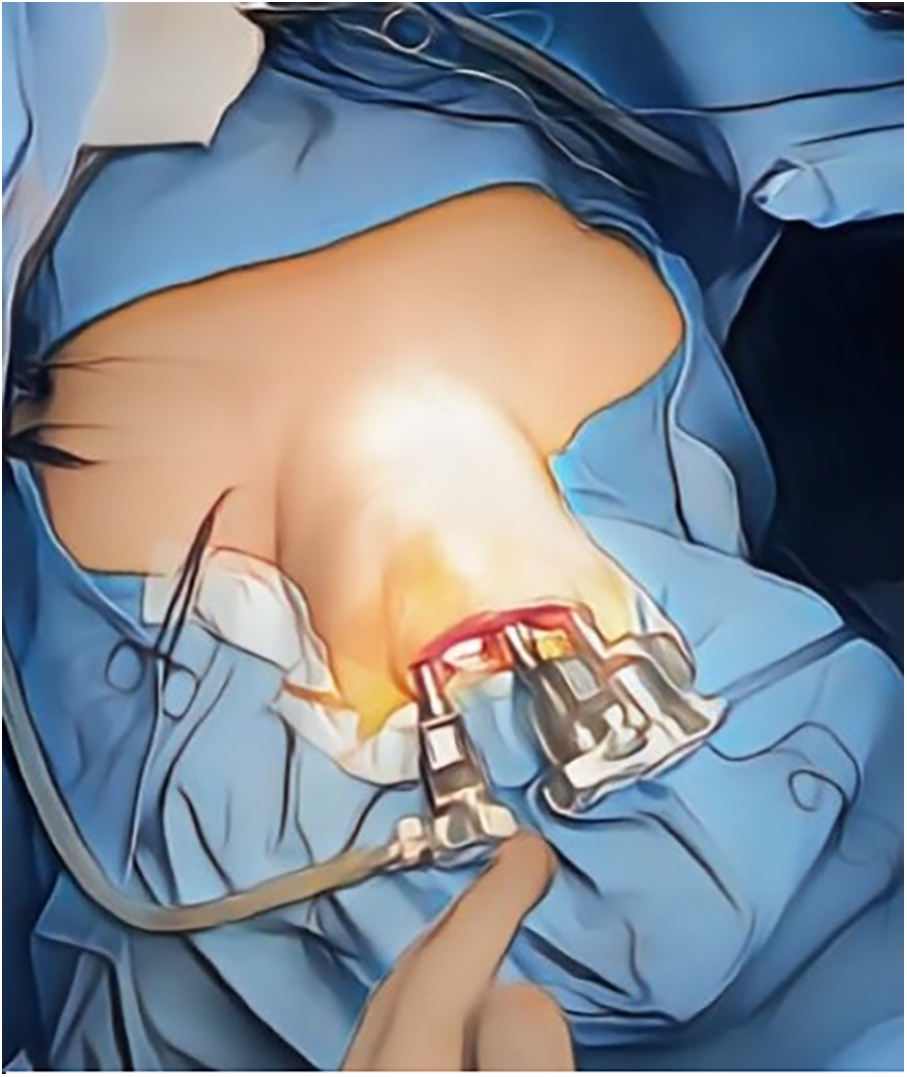
TOETVA procedure.

Before the initiation of the procedure, the patient is carefully evaluated and optimized (24) in order to prevent intraoperative hypotensive events (25, 26). Then, general anesthesia is induced, and the patient is intubated with a specially designed endotracheal tube that allows for intraoperative nerve monitoring. The patient's neck is extended to facilitate the approach to the thyroid gland, and the oral cavity is prepared with antiseptic solution to minimize the risk of infection (15). The TOETVA procedure begins with three incisions: two lateral and one central, all within the lower lip's inner aspect, hidden inside the oral vestibule (6). These incisions are typically less than 1 cm in length and are strategically placed to minimize trauma to oral structures and provide optimal access to the thyroid gland (27). These incisions are used for trocars placement: the 10 mm trocar is central and the two 5 mm trocars are lateral (Figure 1) (28). Blunt dissection is used to create a subplatysmal working space, which is expanded using dilators or insufflation with carbon dioxide to improve visibility and provide room for instrument manipulation. This working space extends from the chin to the sternal notch, and laterally to the sternocleidomastoid muscles on either side. The midline approach helps to minimize the risk of injury to the lateral neurovascular structures (29). The strap muscles are identified and separated along the midline raphe to expose the thyroid gland (16). Careful attention is paid to preserve the integrity and vascular supply of the strap muscles for re-approximation during closure (30). Using laparoscopic instruments through the vestibular incisions, the surgeon begins careful dissection of the thyroid gland (Figure 2) (19). Hemostasis is meticulously maintained using energy devices or clips to control blood vessels (17). The recurrent laryngeal nerve and parathyroid glands are identified and preserved (31). The thyroid lobectomy or total thyroidectomy is performed based on the preoperative indications (32). Dissection of the thyroid lobe began with the pyramidal lobe and continued inferiorly with the division of the isthmus close to the contralateral thyroid lobe using an ultrasonic device (32). After superior pole dissection, the recurrent laryngeal nerve is identified at the insertion and dissected parallel to the trachea and downwards perpendicular to the inferior thyroid artery, near the lower parathyroid gland (33). Then, the thyroid gland was cut close to the thyroid capsule in order to preserve the nerve and lower parathyroid gland (34). If a thyroidectomy is necessary, the same procedure is for the other lobe (35). The indocyanine green (ICG) is useful for locating the parathyroid glands or proofing of the viable glands (36). This could be applied to TOETVA for reducing the rate of hypoparathyroidism (12). Once the thyroid gland or lobes are dissected, they are placed in an endoscopic bag to avoid seeding of the working space and removed through one of the vestibular incisions (37). This may require the gland to be morcellated (cut into smaller pieces) inside the bag, to facilitate removal without enlarging the incision excessively (38). For papillary microcarcinoma patients, central neck lymph node level VI dissection was routinely performed (39). After ensuring complete hemostasis, the strap muscles are reapproximated, and the subplatysmal space is deflated (40). The oral vestibule incisions are then closed with absorbable sutures (41). A drain may be placed in the working space and brought out through the floor of the mouth in some cases, although this practice varies among surgeons (42). A gauze pressure dressing was placed around the chin for 24 h (22). Patients are typically monitored postoperatively for signs of bleeding, infection, or other complications (43). The oral cavity is maintained with antiseptic mouthwash to reduce the risk of infection (44). Postoperative analgesia is managed according to standard protocols (18). The TOETVA procedure requires a significant adjustment in surgical technique compared to traditional cervical thyroidectomy (45). Surgeons must be skilled in endoscopic surgery and familiar with the altered perspective and ergonomics of the transoral approach (13). Modifications to the standard technique may be necessary based on patient anatomy, the extent of the pathology, and intraoperative findings (3).

## 5 Patient selection

TOETVA is primarily indicated for patients with benign thyroid nodules, small to medium-sized goiters, Graves' disease, and in select cases of papillary thyroid carcinoma when the cancer is small (usually less than 2 cm), without evidence of local invasion or distant metastasis (Table 1) (15, 46).

**Table 1:** Indications for TOETVA.

Type of indication	Specific indication
Motivational	Patients must be motivated to avoid a skin incision or an anterior cervical scar
Clinical	History of hypertrophic scarring
	Benign thyroid nodules
	Goiters (small to medium-size)
	Graves disease
	Papillary thyroid carcinoma (no evidence of local invasion or distant metastasis)

Contraindications to TOETVA include, but are not limited to:

• Large goiters that extend substernally or laterally beyond the neck's carotid sheath (47).
• Previous neck surgery or radiation, which could result in altered anatomy and increased risk of complications due to scar tissue (9).
• Advanced or aggressive cancers that involve surrounding tissues and require extensive resection beyond the gland, as reported in other settings (48).
• Coagulopathy or other conditions that predispose patients to bleeding complications, such as anticoagulant therapy (49).
• Morbid obesity or short neck, which may limit the operative working space and make the procedure technically challenging (50).

### 5.1 Preoperative assessment

A comprehensive preoperative assessment is crucial for patient selection:

• Ultrasound Evaluation: To assess the size, location, and characteristics of the thyroid nodules or the extent of the goiter (51).
• Fine-Needle Aspiration (FNA): If indicated, to rule out or confirm malignancy in thyroid nodules (52).
• CT/MRI Scans: To further evaluate the thyroid's anatomy and any substernal extension or surrounding tissue involvement (53).
• Laryngoscopy: To assess vocal cord function before surgery, as any preexisting palsy must be documented (32).

Cosmetic concerns are significant for many patients considering thyroid surgery. TOETVA is particularly suitable for patients who prioritize a scar-free neck, as long as they understand the potential risks and benefits of the procedure compared to conventional methods (4). Patients should be provided with detailed information about the TOETVA, including its novelty, the limited long-term data available compared to traditional approaches, potential risks, and postoperative care (33). Informed consent must be obtained after ensuring that the patient has a clear understanding of the information provided (54). The experience and skill level of the surgeon are also critical factors in patient selection. TOETVA should only be performed by surgeons who have undergone specialized training and have demonstrated proficiency in the technique (44). The healthcare facility must be equipped with the necessary endoscopic equipment and have staff trained in the specific perioperative care requirements of TOETVA patients (19).

## 6 Outcomes and possible complications

The primary measure of surgical success for TOETVA is the complete removal of the targeted thyroid tissue with preservation of vital structures such as the recurrent laryngeal nerves and parathyroid glands (4). Operative time is longer for TOETVA when compared to conventional thyroidectomy, particularly during the initial learning curve (44). As surgeons become more experienced, operative times tend to decrease (15). Complications are a critical metric for any surgical procedure. Despite the minimally invasive nature of the procedure, there is still a risk of bleeding that may require intervention (16). Also, since the oral cavity is not a sterile environment, there is a risk of surgical site infection despite prophylactic antibiotics (6). Moreover, transient or permanent hypoparathyroidism can occur if the parathyroid glands are inadvertently damaged or their blood supply is compromised (28). Lastly, recurrent laryngeal nerve injury and mental nerve injury can lead to temporary or permanent hoarseness or voice changes, as well as numbness or altered sensation in the chin and lower lip (55, 56). Conversion to open surgery is also possible due to difficulties encountered during the procedure (12). Postoperative pain may be less with TOETVA compared to traditional approaches, which can contribute to a quicker recovery and shorter hospital stay (57). Patients often report less discomfort due to the absence of a cervical incision (13). Cosmetic satisfaction is reported to be high among patients who undergo TOETVA, as the procedure leaves no visible neck scars (7). This is particularly significant for patients who place a high value on aesthetic outcomes (58). Patient satisfaction surveys and quality-of-life assessments post-surgery often reflect this positive aspect of the procedure (59). Voice and swallowing function are important considerations in thyroid surgery. TOETVA has been shown to have similar or improved functional outcomes when compared to traditional approaches, although more long-term data is required for a definitive assessment (43). In cases of thyroid cancer, the adequacy of oncologic resection is of utmost concern. A summary of the characteristics of the included studies and the outcomes considered is provided in Table 2. Early studies show promising results, with TOETVA providing similar rates of tumor-free margins compared to conventional thyroidectomy (60). Long-term follow-up is necessary to assess recurrence rates and survival (61). Several studies found the overall incidence of recurrent laryngeal nerve injury of 3.1%–5.9% for TOETVA to be comparable to the incidence of 2.1%–11.8% for open thyroidectomy (15, 62). The low incidence of recurrent laryngeal nerve injury in TOETVA can be explained by the reduced incidence of traction injury and the use of IONM (63). During TOETVA, the recurrent laryngeal nerve is usually identified at its insertion site and released first after division of the Berry's ligament (13). This reduces the risk of traction injury.

**Table 2:** Characteristics of the included studies..

Study	Year	Design	N. TOETVA patients	Outcomes
Chen et al.	2021	Retro PSM	99	1–3, 5–9
Lee et al.	2023	Retro PSM	142	1, 3–6, 8, 9
Anuwong et al.	2018	Retro PSM	216	1, 2, 5–8
Hong et al.	2020	Retro PSM	82	1, 3–9
Han et al.	2020	Retro PSM	50	1, 3, 9
Kasemsiri et al.	2020	Pro CST	32	1, 2, 5, 7, 9, 10
Wang et al.	2020	Retro PSM	80	1–4, 9
Liu et al.	2021	Retro PSM	78	1–10
Sun et al.	2022	Retro PSM	28	1, 3–7, 9
Nguyen et al.	2022	Pro CST	60	1,2,6–10
Li et al.	2023	Retro PSM	101	1–9
You et al.	2021	Retro PSM	186	1, 3–9
Lee et al.	2023	Retro PSM	100	1, 3–7

PSM, retrospective propensity score-matched comparison; CST, prospective cross-sectional study. Outcomes: 1, operative time; 2, blood loss; 3, retrieved lymph nodes; 4, metastatic lymph nodes; 5, recurrent laryngeal nerve palsy; 6, hypoparathyroidism; 7, other complications (including mental nerve injury, surgical site infection, skin flap perforation, chyle leakage, seroma, hematoma, etc.); 8, postoperative pain; 9, postoperative hospital stay; 10, cosmetic satisfaction.

## 7 Future directions

• Technological Advancements

Further development of specialized instruments and endoscopic equipment may enhance the precision and safety of TOETVA. Innovations such as flexible endoscopes or robotic systems with better ergonomics could allow for more delicate dissection and easier manipulation within the confined spaces of the neck.

• Enhanced Visualization

Improvements in visualization technology, such as high-definition cameras and augmented reality (AR), could provide surgeons with more detailed views of the surgical field, potentially reducing the risk of complications and improving surgical accuracy.

• Broadening Indications

As the technique becomes more refined and safety profiles are better understood, the indications for TOETVA may expand. This could include its application in larger goiters, more advanced thyroid cancers, or preoperative surgery in selected cases.

• Training and Education

Establishing standardized training programs and certification processes will be essential to ensure that surgeons are adequately prepared to perform TOETVA. Simulation models, cadaveric workshops, and virtual reality training platforms may become more integral to the educational process.

• Comparative Effectiveness Research

More rigorous comparative studies and randomized controlled trials are needed to evaluate the effectiveness of TOETVA against traditional and other minimally invasive approaches. Long-term outcomes, including recurrence rates in cancer patients and quality of life measures, will be important areas of investigation.

• Patient-Reported Outcomes

Greater emphasis on patient-reported outcomes will help to understand the true impact of TOETVA from the patient's perspective. This includes assessing satisfaction with cosmetic results, pain, recovery time, and overall quality of life after surgery.

• Cost-Effectiveness Analysis

Assessing the cost-effectiveness of TOETVA compared to traditional thyroidectomy and other minimally invasive approaches will be important, considering factors such as operative time, length of hospital stay, and the need for specialized equipment.

• International Collaboration and Registries

Building international collaborations and establishing registries for TOETVA can facilitate the sharing of knowledge, techniques, and outcomes. This global approach can accelerate the accumulation of evidence and help standardize the procedure internationally.

• Genomic and Personalized Medicine

Advancements in genomics and personalized medicine may influence the selection of patients for TOETVA, tailoring the approach based on individual tumor genetics or patient-specific anatomical considerations.

• Artificial Intelligence (AI) and Machine Learning

AI and machine learning algorithms could be developed to assist in preoperative planning, intraoperative decision-making, and postoperative monitoring, potentially improving the precision and outcomes of TOETVA.

• Non-Surgical Alternatives

Research into non-surgical alternatives, such as radiofrequency ablation or percutaneous ethanol injection for benign nodules, may also influence the role of TOETVA in the management of thyroid pathologies.
